# Somatostatin administration prior to ERCP is effective in reducing the risk of post-ERCP pancreatitis in high-risk patients

**DOI:** 10.3892/etm.2014.1733

**Published:** 2014-05-26

**Authors:** LI-NA ZHAO, TAO YU, CHU-QIANG LI, YU LAI, QI-KUI CHEN

**Affiliations:** Department of Gastroenterology, Sun Yat-Sen Memorial Hospital, Sun Yat-Sen University, Guangzhou, Guangdong 510120, P.R. China

**Keywords:** post-endoscopic retrograde cholangiopancreatography pancreatitis, somatostatin, retrospective study, prevention

## Abstract

Somatostatin has been extensively studied for the prophylaxis of pancreatitis following endoscopic retrograde cholangiopancreatography (ERCP). However, the results remain controversial. The present retrospective cohort study aimed to investigate the efficacy of pre- and post-ERCP somatostatin administration in the prevention of post-ERCP pancreatitis (PEP). All ERCP procedures performed at one hospital between January 2009 and December 2012 were reviewed. They were divided into three groups based on somatostatin administration: pre-ERCP som group (somatostatin administration: 0.25 mg/h for 24 h, starting 1 h prior to ERCP), post-ERCP som group (somatostatin administration: 0.25 mg/h for 24 h, starting immediately following ERCP), and control group (no somatostatin administration). Out of a total of 304 cases, 81 received pre-ERCP somatostatin; 126 received post-ERCP somatostatin and 97 were not administered somatostatin. Pre-ERCP somatostatin was effective in reducing the incidence of PEP compared with that in the control group (4.9 vs. 16.5%; P=0.017). This benefit was significant in high-risk patients (8.9 vs. 26.0%; P=0.035), but not in low-risk patients (0 vs. 6.4%; P=0.254). Post-ERCP somatostatin was not effective in preventing PEP in high- or low-risk patients. In conclusion, pre-ERCP somatostatin may be effective in reducing the risk of PEP in high-risk patients, but not in low-risk patients. Post-ERCP somatostatin did not reveal a benefit in high- or low-risk patients. However, large randomized controlled trials are required to further confirm these findings.

## Introduction

Endoscopic retrograde cholangiopancreatography (ERCP) is a widely available and routine procedure for treating a wide variety of pancreaticobiliary diseases. Due to its advantage of being a minimally invasive treatment, it is generally accepted as a valuable and promising therapeutic modality, especially in choledocholithiasis (1). However, a high frequency of complications is associated with the ERCP procedure. Post-ERCP pancreatitis (PEP) is the most common complication, with an incidence rate ranging from 1 to 15.1% of cases, rising to 30% in high-risk patients (2).

Numerous mechanisms have been proposed for the development of PEP (3). A common mechanism involves an initial pancreatic injury that leads to the premature activation of proteolytic enzymes. This initiates an inflammatory cascade that results in local and systemic effects. Two factors may lead to the initial pancreatic injury. One is the obstruction of pancreatic outflow caused by mechanical-, chemical-, or electrocautery-related thermal injury and consequent edema during cannulation and instrumentation of the papilla. The other factor is the increased hydrostatic pressure resulting from pancreatic duct injection (3).

Numerous methods, including ERCP techniques and pharmacological agents, have been introduced in an attempt to prevent PEP. Among the ERCP techniques, pancreatic duct stenting has been recommended to reduce PEP in high-risk patients (4). However, the challenging placement technique and the necessity for follow-up evaluations to ensure passage or removal have limited its clinical application. Therefore, various drugs have been considered that could be used to prevent PEP. Among these, somatostatin was identified as one of the most promising drugs and has thus been extensively studied. However, the results remain controversial (5–13). In the European Society of Gastrointestinal Endoscopy (ESGE) guidelines, prophylactic somatostatin administration is not recommended for average-risk patients. However, the administration of somatostatin may be more effective in high-risk patients or when administered using specific dose schedules (4). Therefore, further investigation is required to uncover appropriate methods of using prophylactic somatostatin for PEP prevention.

The present retrospective cohort study was conducted to investigate the efficacy of pre- and post-ERCP somatostatin administration in the prevention of PEP.

## Patients and methods

### Patients and grouping

All ERCP procedures between January 2009 and December 2012 at the Sun Yat-Sen Memorial Hospital (Guangzhou, China) were reviewed. Hospitalized patients who had undergone ERCP procedures were included in the current study. The exclusion criteria included: i) hyperamylasemia at the baseline blood test; ii) acute pancreatitis prior to ERCP; and iii) failed cannulation.

Based on the different methods of somatostatin administration used at the hospital to prevent PEP, the patients were classified into the pre-ERCP som group, receiving somatostatin 1 h prior to ERCP by continuous intravenous infusion (0.25 mg/h) for 24 h; the post-ERCP som group, receiving somatostatin immediately following ERCP by continuous intravenous infusion (0.25 mg/h) for 24 h; and the control group, not receiving somatostatin administration. The patient demographics, indications, interventions, method of somatostatin administration and complications were noted for further analysis. Informed consent was obtained from the patients or the patients’ families. The present study was approved by the ethics committee of Sun Yat-Sen Memorial Hospital.

### ERCP procedures

All patients were fasted for 12 h prior to and at least 12 h following surgery. Intravenous sedation with meperidine and midazolam was given during the procedure. One dose of intravenous prophylactic antibiotic (cefoperazone 1 g) and proton pump inhibitor were given following the procedure. Selective cannulation of the common bile duct was attempted in all patients and pancreatic duct cannulation was attempted only if indicated. Sphincterotomy and therapeutic procedures including stone extraction (balloon, basket or mechanical lithotripsy) and bile duct stenting (plastic stent) were performed when indicated. All ERCP procedures carried out in the present study were performed by the same experienced endoscopist. A difficult cannulation was defined as ≥3 cannulation attempts. Details of the endoscopic procedure were recorded by the endoscopist immediately following the procedure.

### Post-ERCP monitoring

All patients remained in the hospital for ≥72 h following ERCP. Serum amylase levels were routinely measured prior to ERCP (baseline) and at 6 and 24 h following ERCP. Patients’ symptoms, including abdominal pain and tenderness, were also documented during the 24 h following ERCP.

### PEP and hyperamylasemia definitions

PEP was defined as new or worsened abdominal pain and tenderness persisting for >24 h following ERCP, with an elevated serum amylase level >3 times the normal upper limit. Based on previous studies, the severity of pancreatitis was classified as mild when the length of hospital stay was ≤3 nights, moderate when the hospital stay was 4–10 nights, and severe if >10 days of hospitalization, intensive care unit admission, or surgery were required for the pancreatitis (14). The length of hospital stay was the number of days from the date of ERCP surgery until the date of the resolution of abdominal pain and reduction of serum amylase levels to <2-fold higher than the normal upper limit. The second outcome, hyperamylasemia, was defined as an elevation in serum amylase levels to at >2-fold higher the upper normal limit at 6 or 24 h following ERCP.

### High- and low-risk patient definitions

Following careful consideration and discussion of various previous studies (4,15), patients who satisfied one of the following risk factors of PEP were defined as high-risk patients in the current study: i) suspected sphincter of Oddi dysfunction (SOD), defined as a pre-ERCP suspicion of a functional or structural abnormality of the sphincter of Oddi, independent of any manometric findings, considered to be the potential cause of recurrent abdominal pain or pancreatitis; ii) recent acute pancreatitis; iii) precut sphincterotomy; iv) difficult cannulation (cannulation attempted ≥3 times); and v) pancreatic duct injection (4,15). Patients with none of the above risk factors were defined as low-risk patients.

### Statistical analysis

Variables were reported using means (with standard deviations) and simple proportions. Analysis was performed using analysis of variance (ANOVA) and the χ^2^ test (or Fisher’s exact test when appropriate). If a two-tailed P-value was <0.05 among the three groups, then further analysis was carried out to compare pre- or post-ERCP groups with the control group. Multivariate (logistic regression) analysis was also carried out to further confirm the effect of somatostatin on preventing PEP and to analyze the risk factors of PEP at the hospital. The data was analyzed with SPSS software version 13.0 (SPSS, Inc., Chicago, IL, USA). A two-tailed P-value <0.05 was considered to indicate a statistically significant results.

## Results

### Patient data

There was a total of 343 ERCP cases from January 2009 to December 2012 at the hospital. Of these, 39 were excluded from further analysis: 25 due to serum amylase levels being higher than the normal upper limit prior to ERCP, 4 due to acute pancreatitis prior to ERCP and 10 due to failed cannulation. In the 304 enrolled cases, 81 cases received somatostatin 1 h prior to ERCP (pre-ERCP som group), 126 cases received somatostatin administration immediately following ERCP (post-ERCP som group) and the remaining 97 cases with no somatostatin administration were the control group.

Table I shows patient characteristics, ERCP indications and procedures. Overall, biliary ductal stones were identified as an indication of ERCP in 71.4% of the patients. No statistically significant differences in mean age, gender, SOD and ERCP indications were identified among the three groups. Among the procedures, pancreatic duct stenting was performed on only 8.2% of cases (11.1% in the pre-ERCP som group, 7.9% in the post-ERCP som group and 6.2% in the control group). The proportions of patients undergoing difficult cannulation, precut sphincterotomy, pancreatic duct injection, biliary sphincterotomy, stone extraction, drain insertion and pancreatic duct stenting procedures were similar among the three groups.

### Incidences of PEP and hyperamylasemia

Among the 304 included ERCP cases, the overall incidences of PEP and hyperamylasemia were 12.8% (39/304) and 27.0% (82/304), respectively. Most PEP was mild and mild PEP occurred in 9.5% of cases. Moderate PEP occurred in 3.3% of cases (none in the pre-ERCP som group, 4.0% in the post-ERCP som group and 5.2% in the control group). No severe PEP occurred. Statistical analysis revealed that the incidence of PEP in the pre-ERCP som group was significantly lower than that in control group (4.9 vs. 16.5%; P=0.017; Table II). However, somatostatin administration immediately following ERCP (post-ERCP som group), did not demonstrate the same benefit when compared with the control group (15.1 vs. 16.5%; P=0.853; Table II). Neither somatostatin administration in the pre-ERCP som group nor in the post-ERCP som group was able to reduce the incidence of hyperamylasemia compared with that in the control group (P=0.231; Table II).

### Incidences of PEP and hyperamylasemia in high- or low-risk patients

Of the 304 patients in this study, 160 were defined as high-risk and 144 as low-risk. Among high-risk patients, the overall incidences of PEP and hyperamylasemia were 21.3% (34/160) and 34.4% (55/160), respectively. The incidence of PEP in high-risk patients in the pre-ERCP som group was significantly lower than in high-risk patients in the control group (8.9 vs. 26.0%; P=0.035; Table III); however, the difference was not significant between the post-ERCP som group and the control group (26.2 vs. 26.0%; P=0.985; Table III). In low-risk patients, the overall incidences of PEP and hyperamylasemia were 3.5% (5/144) and 25.7% (37/144), respectively. The incidences of PEP among the pre-ERCP som group, post-ERCP som group and control group were similar (0, 3.3, and 6.4%, respectively; P=0.371; Table IV). The incidence of hyperamylasemia in high-risk patients was not significantly different among the three groups (P=0.168; Table III), nor was it significantly different in the low-risk patients (P=0.858; Table IV).

### Factors associated with PEP

To assess the independent role of the effect of somatostatin on PEP, logistic regression analysis for all the independent factors associated with PEP identified in previous studies (female gender, recent acute pancreatitis, SOD, difficult cannulation, precut sphincterotomy, pancreatic duct injection, biliary sphincterotomy, pancreatic duct stenting, pre-ERCP somatostatin administration and post-ERCP somatostatin administration) was carried out (15). Pre-ERCP somatostatin administration was significantly associated with PEP reduction [odds ratio (OR) 0.17; 95% confidence interval (CI) 0.05–0.62; P=0.007; Table V]. Pancreatic duct stenting was also identified as a protective factor for PEP (OR 0.15; 95% CI 0.03–0.78; P=0.024; Table V). The factors SOD, difficult cannulation and pancreatic duct injection were identified as risk factors of PEP.

## Discussion

ERCP is an effective and irreplaceable method for the diagnosis and treatment of pancreaticobiliary diseases. PEP is a common complication of ERCP with an incidence rate ranging from 1 to 15.1% of cases, which may increase to 30% in high-risk patients. Most PEP is relatively mild and the main consequence is prolonged hospital stay and increased healthcare expenditure. However, in rare cases (~0.3–0.6%) severe pancreatitis may be life threatening and have a devastating impact on the patient’s quality of life (2). Consistent with previous studies, the results of the present study indicated that the overall frequency of PEP was 12.8% in all patients, 21.3% in high-risk patients and 3.5% in low-risk patients.

With many positive biological effects, including inhibition of exocrine pancreatic secretion, reduction of sphincter of Oddi contractions, modulation of the cytokine cascade and possible pancreatic acinar cytoprotection, somatostatin was believed to be one of the most promising drugs that could be used to reduce the risk of PEP (16–18). A meta-analysis of 10 randomized controlled trials (RCTs) concluded that somatostatin was able to reduce the risk of PEP (RR 0.52; 95% CI 0.30–0.90), especially in cases of pancreatic duct injection, biliary sphincterotomy, high dose infusion over 12 h, and bolus injection (19). However, in the ESGE guidelines, a meta-analysis of another 10 high-quality trials indicated that somatostatin administration did not result in a reduction of PEP (OR 0.57; 95% CI 0.32–1.03). It further stated, however, that when the baseline incidence of PEP among the control group was >10%, a benefit to somatostatin administration was observed, and an infusion of somatostatin for >12 h may improve its efficacy (4). In the current study, somatostatin administered 1 h prior to ERCP by continuous intravenous infusion (0.25 mg/h) for 24 h reduced the incidence of PEP (Table II). Further subgroup analysis indicated that this benefit was significant in high-risk patients but not in low-risk patients (Tables III and IV). Multivariate analysis also confirmed that pre-ERCP somatostatin was effective in reducing PEP (OR 0.17; 95% CI 0.05–0.62; P=0.007; Table V). These data suggest that pre-ERCP somatostatin administration may be effective in reducing the risk of PEP in high-risk cases.

In the majority of previous studies, somatostatin infusion was performed 30 min or 1 h prior to ERCP. The administration of somatostatin at these times makes it difficult to predict the risk factors associated with the procedure, including precut sphincterotomy, difficult cannulation, pancreatic duct injection and pancreatic sphincterotomy. As it is difficult to evaluate all the risk factors when somatostatin is administered exclusively as a prophylactic treatment prior to ERCP in high-risk cases, post-ERCP somatostatin was also studied. A RCT of 270 cases carried out by Poon *et al* revealed that the incidence of PEP was significantly lower in the group with somatostatin administration immediately following diagnostic ERCP but prior to therapeutic ERCP, when compared with that in the placebo group (4.4 vs. 13.3%; P=0.01) (11). Another RCT carried out by Wang *et al* suggested that somatostatin administration 1 h following ERCP was effective in preventing hyperamylasemia in ERCP cases, but not in reducing PEP (20). The results of the present study suggest that somatostatin administration immediately following ERCP is an ineffective method of reducing PEP and hyperamylasemia, even in high-risk patients. A possible reason for this may be that the development of PEP is a process in which the inflammatory cascade reaction is initiated by pancreatic injury during ERCP. Thus, avoiding initial pancreatic injury is pivotal to preventing PEP. However, post-ERCP somatostatin administration is not able to reduce the initial pancreatic injury during the ERCP procedure. Therefore, earlier somatostatin administration is pivotal to reducing PEP and somatostatin administration prior to ERCP may be inevitable. However, this requires further investigation.

According to numerous prospective studies, the risk factors of PEP include patient-related risk factors (young age, female gender, recent acute pancreatitis and SOD) and procedure risk factors (precut sphincterotomy, difficult cannulation, pancreatic duct injection, and pancreatic sphincterotomy) (4). The present study also analyzed the risk factors of PEP using multivariate analysis. Consistent with previous studies, SOD, difficult cannulation and pancreatic duct injection were identified to be independent risk factors of PEP (Table V). However, certain known risk factors (gender, recent acute pancreatitis, and precut sphincterotomy) were not identified as risk factors of PEP in the current study. The major indicators of ERCP in the present study were biliary ductal stones. A study by Testoni *et al* demonstrated that in patients undergoing ERCP for biliary ductal stones, precut sphincterotomy did not appear to be an independent risk factor for PEP (21). Nevertheless, larger prospective studies are required to confirm these results.

Although the present study revealed that pre-ERCP somatostatin administration was an effective method of reducing the risk of PEP whereas post-ERCP somatostatin administration was not, the results have limitations. Firstly, the data collection was retrospective. Although the primary factors associated with PEP were accurately collected, potential bias was inevitable. Secondly, all the data was collected from medical records which may have contained inaccurate information. However, since the method of somatostatin administration was a pivotal factor for its efficacy in preventing PEP, the current study revealed that somatostatin administration 1 h prior to ERCP of 0.25 mg/h for 24 h was effective in reducing PEP in high-risk patients, but not in low-risk patients.

In conclusion, somatostatin administration prior to ERCP may be effective in reducing the risk of PEP in high-risk patients, but not in low-risk patients. Somatostatin administration immediately following ERCP did not demonstrate this benefit in either high- or low-risk patients. However, large RCTs are required in order to confirm these results.

## Figures and Tables

**Table I:** Baseline characteristics among the three groups.

Characteristic	Pre-ERCP som (n=81)	Post-ERCP som (n=126)	Control (n=97)	P-value
Age (years; mean ± SD)	59.31±14.61	56.06±14.38	56.23±15.94	0.262
Gender (female/male)	41/40	58/68	52/45	0.519
Indications [n (%)]				
Biliary ductal stones	59 (72.8)	92 (73.0)	66 (68.0)	0.684
Recent acute pancreatitis	9 (11.1)	8 (6.3)	7 (7.2)	0.438
Malignancy	5 (6.2)	12 (9.5)	11 (11.3)	0.503
Others	8 (9.9)	14 (11.1)	13 (13.4 )	0.764
SOD [n (%)]	15 (18.5)	27 (21.4)	14 (14.4)	0.414
Difficult cannulation^a^ [n (%)]	16 (19.8)	17 (13.5)	12 (12.4)	0.349
Precut sphincterotomy [n (%)]	14 (17.3)	12 (9.5)	11 (11.3)	0.221
Pancreatic duct injection [n (%)]	26 (32.1)	44 (34.9)	34 (35.1)	0.906
Biliary sphincterotomy [n (%)]	66 (81.5)	100 (79.4)	76 (78.4)	0.888
Stone extraction [n (%)]	59 (72.8)	91 (72.2)	65 (67.0)	0.635
Drain insertion^b^ [n (%)]	10 (12.3)	18 (14.3)	21 (21.6)	0.206
Pancreatic duct stenting [n (%)]	9 (11.1)	10 (7.9)	6 (6.2)	0.501

a Difficult cannulation (≥3 cannulation attempts);

b drain insertion includes biliary stenting and nasobiliary drainage.

SOD, sphincter of Oddi dysfunction; pre-ERCP som, pre-endoscopic retrograde cholangiopancreatography somatostatin administration; post-ERCP som, post-endoscopic retrograde cholangiopancreatography somatostatin administration.

**Table II:** Clinical outcomes of patients in the three groups.

Clinical outcome	Pre-ERCP som [n=81; n (%)]	Post-ERCP som [n=126; n (%)]	Control [n=97; n (%)]	P-value
PEP	4 (4.9)	19 (15.1)	16 (16.5)	0.032^a^
Mild	4	14	11	
Moderate	0	5	5	
Severe	0	0	0	
Hyperamylasemia	24 (29.6)	38 (30.2)	20 (20.6)	0.231

a Pre-ERCP som vs. control: P=0.017, post-ERCP som vs. control: P=0.853.

PEP, post-endoscopic retrograde cholangiopancreatography pancreatitis; pre-ERCP som, pre-endoscopic retrograde cholangiopancreatography somatostatin administration; post-ERCP som, post-endoscopic retrograde cholangiopancreatography somatostatin administration.

**Table III:** Clinical outcomes of high-risk patients in the three groups.

Clinical outcome	Pre-ERCP som [n=45; n (%)]	Post-ERCP som [n=65; n (%)]	Control [n=50; n (%)]	P-value
PEP	4 (8.9)	17 (26.2)	13 (26.0)	0.045^a^
Hyperamylasemia	18 (40.0)	25 (38.5)	12 (24.0)	0.168

a Pre-ERCP som vs. control: P=0.035; post-ERCP som vs. control: P=0.985;

PEP, post-endoscopic retrograde cholangiopancreatography pancreatitis; pre-ERCP som, pre-endoscopic retrograde cholangiopancreatography somatostatin administration; post-ERCP som, post-endoscopic retrograde cholangiopancreatography somatostatin administration.

**Table IV:** Clinical outcomes of low-risk patients in the three groups.

Clinical outcome	Pre-ERCP som [n=36; n (%)]	Post-ERCP som [n=61; n (%)]	Control [n=47; n (%)]	P-value
PEP	0	2 (3.3)	3 (6.4)	0.371^a^
Hyperamylasemia	16 (16.7)	13 (21.3)	8 (17)	0.858

a Pre-ERCP som vs. control: P=0.254, Post-ERCP som vs. control: P=0.651;

PEP, post-endoscopic retrograde cholangiopancreatography pancreatitis; pre-ERCP som, pre-endoscopic retrograde cholangiopancreatography somatostatin administration; post-ERCP som, post-endoscopic retrograde cholangiopancreatography somatostatin administration.

**Table V:** Factors associated with PEP in the multivariate analysis.

Factor	PEP cases (n=39)	Control (n=265)	OR (95% CI)	P-value
Female	19	151	0.85 (0.38–1.90)	0.688
Recent acute pancreatitis	3	24	0.87 (0.20–3.89)	0.872
SOD	15	56	4.70 (1.86–11.89)	0.001
Difficult cannulation^a^	14	45	5.76 (1.75–19.02)	0.004
Precut sphincterotomy	9	37	0.45 (0.12–1.75)	0.249
Pancreatic duct injection	26	104	4.37 (1.93–9.93)	<0.001
Biliary sphincterotomy	35	242	2.38 (0.71–8.05)	0.162
Pancreatic duct stenting	3	25	0.15 (0.03–0.78)	0.024
Pre-ERCP som	4	81	0.17 (0.05–0.62)	0.007
Post-ERCP som	19	126	0.72 (0.31–1.64)	0.431

a difficult cannulation (≥3 cannulation attempts);

SOD, sphincter of Oddi dysfunction; PEP, post-endoscopic retrograde cholangiopancreatography pancreatitis; pre-ERCP som, pre-endoscopic retrograde cholangiopancreatography somatostatin administration; post-ERCP som, post-endoscopic retrograde cholangiopancreatography somatostatin administration; OR, odds ratio; CI, confidence interval.
